# Insight into the active site nature of zeolite H-BEA for liquid phase etherification of isobutylene with ethanol

**DOI:** 10.1039/c9ra07721a

**Published:** 2019-11-05

**Authors:** Nina V. Vlasenko, Yuri N. Kochkin, German M. Telbiz, Oleksiy V. Shvets, Peter E. Strizhak

**Affiliations:** L. V. Pisarzhevskii Institute of Physical Chemistry, National Academy of Sciences of Ukraine Prospect Nauki 31 03039 Kiev Ukraine nvvlasenko@gmail.com

## Abstract

The nature of active acid sites of zeolite H-BEA with different Si/Al ratios (15–407) in liquid phase etherification of isobutylene with ethanol in a continuous flow reactor in the temperature range 80–180 °C has been explored. We describe and discuss data concerning the strength and concentration of acid sites of H-BEA obtained by techniques of stepwise (quasi-equilibrium) thermal desorption of ammonia, X-ray diffraction, low-temperature adsorption of nitrogen, FTIR spectroscopy of adsorbed pyridine and solid-state ^27^Al MAS NMR. The average values of the adsorption energy of NH_3_ on H-BEA were experimentally determined as 63.7; 91.3 and 121.9 mmol g^−1^ (weak, medium, and strong, respectively). In agreement with this, a correlation between the rate of ethyl-*tert*-butyl ether synthesis and the concentration of weak acid sites (*E*_NH_3__ = 61.6–68.9 kJ mol^−1^) has been observed. It was concluded that the active sites of H-BEA for this reaction are Brønsted hydroxyls representing internal silanol groups associated with octahedrally coordinated aluminum in the second coordination sphere.

## Abstract 2

The active sites of H-BEA zeolites for ETBE synthesis are the weak Brønsted acid sites representing internal silanol groups.

## Graphical abstract

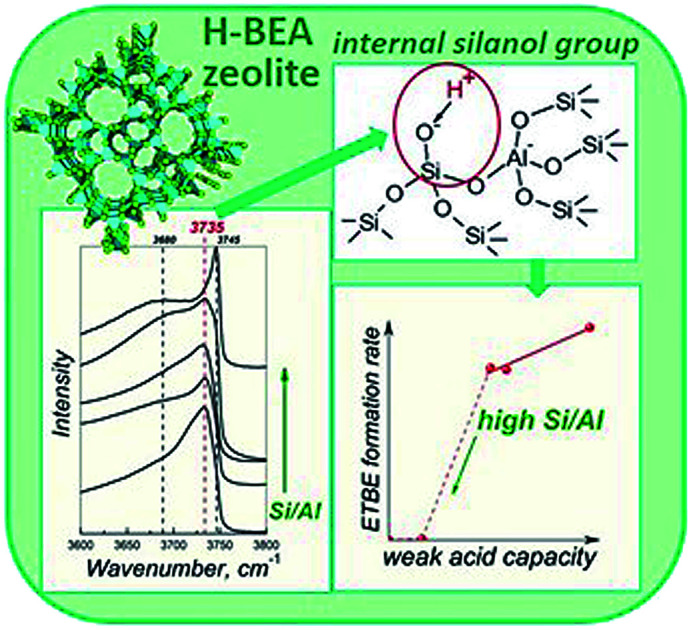

## 1. Introduction

Zeolites are the most important solid acids as highly active and selective catalysts in a number of commercial chemical processes.^1–4^ The acidity allows protonic zeolites to have widespread application as solid catalysts in a large range of chemical processes, *e.g.*, catalytic cracking,^5–7^ isomerization,^8–10^ and alkylation.^11–13^ The catalytic activity of zeolites is mainly associated with the Brønsted acid and (or) Lewis acid sites, and a result of the replacement of Al for Si in the tetrahedral units, which generates a net negative charge that has to be balanced by extra framework cations or protons. The concentration of acid sites depends on the Si/Al ratio and their acid strength is believed to be dependent on the structure of the three-dimensional network as well as on the local atomic environment of the site.^14^ The active sites of zeolites are situated in pores and channels of different dimensions as well as on the external surface of the microcrystals in the catalytic bed. Depending on their location, acid sites in zeolites might be hidden and unavailable to participate in catalytic reactants. The recent developments and trends in tailoring the nature and local properties of active sites in zeolite-based catalysts were reviewed in ref. 15 and 16.

The catalytic performance of zeolites depends in a different way on the strength, concentration, and structure of their Brønsted acid sites.^17–19^ The concentration and strength of the acid sites in zeolites constitutes a main factor determining the catalytic activity and the selectivity of zeolites, and hence, a reliable method to quantify acidity is an importance. Several instrumental techniques can be used for this purpose; among them the ammonia thermal desorption (NH_3_ TPD) are typically used.^20–23^ High basicity and low size of ammonia molecule allow characterizing all acid sites independently on their nature, localization, and strength. Other experimental techniques are informative for evaluating the acid properties of the hydroxyl groups in zeolites, *e.g.*, calorimetric measurements of simple probe molecules,^24^ titration of the surface with Hammett indicators,^25,26^^27^Al MAS NMR,^27–29^^13^C NMR chemical shifts of adsorbed acetone,^30^ FTIR spectroscopic studies,^31–37^ ammonia infrared/mass spectroscopy (IRMS) temperature-programmed desorption (TPD).^38,39^

Recently, large pore HBEA zeolites became a subject of numerous studies in catalysis. Zeolite β (BEA) has a three-dimensional channel system with 12-membered ring apertures of 5.6–7.3 Å and high external surface area.^40,41^ Zeolite BEA is more active than other zeolites especially if the processing of bulk molecules is needed. The small crystal size of BEA zeolites (20–50 nm) causes a short diffusion path of reactants and products, and therefore transport limitations do not play perceptible role in the catalytic reactions.^42^ Due to the successful combination of structural and acid characteristics, namely, open pore structure and high acidity,^43,44^ the protonic form of BEA is an active solid acid catalyst for many kinds of industrially important reactions, like acylation,^45,46^ alkylation,^47–49^ esterification,^45,50,51^ etherification,^52,53^ isomerization,^54,55^ dehydration^56,57^*etc.*

In last decades the etherification of alcohols, especially of bioethanol which is considered as a renewable crude, becomes important commercial processes for the production of a variety of oxygenates. Oxygenated compounds are known as octane improvers for liquid fuels.^58,59^ Their use as additives to fuel reduces the exhaust of carbon monoxide and unburned hydrocarbons, minimizing the emission of volatile organic compounds.^60,61^ In industry, alkyl *tert*-butyl ethers are produced using various sulfonic cation-exchange resins as catalysts.^62^ They are characterized by high activity and selectivity, however, they have certain drawbacks: low mass exchange characteristics, poor heat resistance, and tendency to swell under the action of polar reagents, which causes technological disadvantages of their practical use.

The worldwide production and consumption of the most popular fuel additive MTBE have sharply decreased for environmental reasons, whereas production of oxygenates from bio-resources, particularly, ethyl *tert*-butyl ether (ETBE), is growing. ETBE exhibits higher octane rating, higher boiling point, lower flash point, lower blending Reid vapor pressure, and reasonably high oxygen contents.^63,64^ Moreover, the attraction of the ETBE industrial application is defined by the possibility to use bioethanol, and also to obtain gasoline with reduced volatility.

Various acid catalysts have been studied in ETBE synthesis from ethanol and isobutylene, particularly, zeolites,^65,66^ macroporous sulfonic acid resins,^67,68^ and heteropolyacids.^69^ ETBE synthesis is commercially operated in the liquid phase over various types of acidic ion-exchange resins.^70^ Typically, these catalysts are presented as sulfonated resins with styrene–divinylbenzene matrix. The catalytic efficiency of sulfonic acid groups located both on the polymer surface and inside the grain is governed by their accessibility to molecules of reagents. Therefore, both the mass-transfer process and the adsorption–desorption dynamics should be important factors influencing the catalytic behavior in etherification processes. These properties are determined by morphology, porous structures and size of catalyst grain.^71^ Decreasing the optimum temperature of the ETBE synthesis with simultaneous increase of efficiency has appeared possible under the condition of maintaining the accessibility of zeolite acid sites.^72–75^

Usually, the activity of zeolites catalyst in acid-catalyzed reactions binds to the total amount of acid sites. In ETBE synthesis over zeolite beta with different Si/Al, it was found that the yield at maximum isobutylene conversion is proportional to the external surface area.^66^ However, the role of strong and weak acid sites in a catalytical transformation of the reactants on BEA surface has not been clarified.

Impact of weak acid sites of zeolites on the catalytic behaviors has gathered a lot of attention, mainly as it was considered in the aspect of their non-acidity. However, recently it has been shown that external silanols in zeolites have weak acidic nature.^76,77^ The significance of weak acid surface groups in some catalytic reactions occurring in soft conditions was reported.^78,79^ The good performance of zeolite BEA in MTBE synthesis from methanol and isobutylene showed activity comparable to the commercial ion-exchange resin Amberlyst-15 that has been explained as a result of the adsorption of methanol on the silanols of the zeolite BEA.^80^ Contrary, zeolites HY and HZSM-5 showed substantially lower activity in the MTBE synthesis due to the methanol is mainly adsorbed on the bridging OH groups.

Another example of studying role of weak acid sites is presented by the effect of zeolites acidity on their catalytic activity in etherification of 2-naphthol with ethanol over H-beta, H-MOR and H-ZSM-5.^81^*t* was expected that the etherification activity of zeolites may be affected by the weak acid sites. In cyclohexanone oxime Beckmann rearrangement reaction MgSiAlPO-5 molecular sieve having weak acidity demonstrated considerably superior performance than the relatively stronger acidic MgAlPO-5 catalyst.^82^

It has been found that H-BEA zeolites with Si/Al ratios of 12.2 and 36 were more active in the MTBE synthesis than industrial catalyst Amberlyst-15 at temperatures between 40 and 100 °C.^80^ The most active H-BEA sample with Si/Al = 36 contains a higher concentration of silanols which contribute to the adsorption of the alcohol. However, no correlation between acid and the catalytic characteristics was found.

In our previous work, we have established a crucial role of the weak acid sites of zeolites for their catalytic behavior in the ETBE synthesis.^83^ However, the nature of these sites was not clear. In this study we present the results to highlight the nature of the weak acid sites of H-BEA zeolites which are active in the etherification of isobutylene with ethanol. For this purpose, H-BEA zeolites with a wide range of Si/Al ratios were studied. The ammonia Quasi-Equilibrium ThermoDesorption (QETD) has been chosen to characterize both concentration and strength of acid sites. Comparison of these data with results of the catalytic tests allows us to establish a relationship between acidic and catalytic characteristics for H-BEA zeolites. Moreover, these results highlight an effect of the Si/Al ratio on the catalytic performance of zeolite H-BEA. These data supported by FTIR spectroscopy of adsorbed pyridine and solid-state ^27^Al MAS NMR gives an understanding of the nature and structure of the weak acid sites which are active in the etherification of isobutylene with ethanol.

## 2. Experimental

### 2.1. Materials

Commercially available ethanol (azeotropic mixture with 4.43 wt% H_2_O), isobutylene, and helium were used as purchased without additional purification. For investigation of catalysts acidity commercially available ammonia was dried by passing through the trap with NaA zeolite.

### 2.2. Catalysts preparation

The synthesis of the BEA zeolite using hydrothermal template synthesis was performed as follows. In typical synthesis, solution containing 0.044 g of LiOH and 0.149 g of Al(NO_3_)_3_ were dissolved in 6.7 ml of deionized water and were mixed with 6.51 ml of 35% tetraethylammonium hydroxide. The mixture was maintained at room temperature for 30 min and then dropwise added 2.83 ml of colloidal solution SiO_2_ (Ludox HS-40). The reaction mixture was transferred into a Teflon-lined stainless steel autoclave and hydrothermally treated at 150 °C for 4 days. Resulting product was filtered, washed with distilled water, dried at 80 °C for 10 h and calcined at 550 °C for 5 hours. H-BEA zeolites were obtained by ion exchange with 1 M NH_4_NO_3_ aqueous solution at 80 °C under stirring. The resulting solid was filtered, washed with deionized water, dried in air at 100 °C for 10 h, and calcined at 450 °C for 5 h. The different Si/Al ratio in samples was regulated by changing of SiO_2_/Al_2_O_3_ molar ratio of in reaction mixture. Marking of samples corresponds to the Si/Al ratio in zeolites.

### 2.3. Sample characterization

The silicon and aluminum content was determined by standard methods with an error less than 2%.^84,85^ Crystallinity and phase purity of solids were determined by XRD. XRD patterns were recorded in the 2*θ* range of 5–50° with a scan speed of 2° min^−1^ on a Bruker AXS D8 ADVANCE diffractometer in the Bragg–Brentano geometry using CuK_α_ radiation (*λ* = 0.1542 nm, 40 kV, 20 mA) with a NaI dynamic scintillation detector. Nitrogen adsorption isotherms were performed at −196 °C in the 0.001–1 relative pressure range using Sorptomatic 1990 apparatus (Thermo Electron Corporation). Prior to the adsorption measurements, all samples were degassed in flowing N_2_ for 5 h at 300 °C. The surface area (*S*_BET_) was calculated by the standard BET method using nitrogen adsorption data in the relative pressure range from 0.01 to 0.10 because deviations from linearity of the BET plot were observed at relative pressures below 0.01 and above 0.10. The micropore volume (*V*_mi_) was calculated from the cumulative pore size distribution as the volume of pores with sizes less than 2 nm.

^27^Al Magic Angle Spinning Nuclear Magnetic Resonance (MAS-NMR) spectroscopy measurements were carried out using an “Avance 400” (“Bruker”) spectrometer equipped with a multinuclear probe, with 4 mm zirconia rotors spinning at 10 kHz. A single pulse sequence was used, with a pulse length of 15 ms, an observation frequency for ^27^Al of 52.12 MHz. The chemical shifts (*δ*, ppm) are reported in ppm from 0.1 M AlCl_3_ solution.

### 2.4. Acidity measurement

Acid properties of samples were investigated by Quasi-Equilibrium Thermodesorption (QETD) of ammonia.^78,83^ Experiments were carried out at 50–500 °C under vacuum (constant pressure 0.133 Pa was maintained by vacuum pump) using thermogravimetric apparatus with quartz spring balance of McBain type. Prior to the experiment, the sample was outgassed at the temperature of its calcination during a time sufficient to reach a constant weight of the sample (typically 1.5–2 h). Then, the temperature was lowered to ambient conditions and ammonia was admitted into sample stepwise (10–20 torr) until no uptake was observed. Finally, an excess of ammonia was evacuated at 50 °C. The temperature was raised stepwise (5 °C min^−1^ between steps, step size 50 °C). After raising, the temperature was kept fixed until constant weight was achieved (typically 10–15 min). As a result, experimental conditions corresponded to desorption at constant pressure, *i.e.* desorption isobar. The concentration of each type of acid sites was calculated from the amount of ammonia sorption (mmol g^−1^) in a certain temperature range determined by the form of the QETD curve. The total concentration of the acid sites (acid capacity) was determined as a quantity of ammonia adsorbed on a sample surface at 50 °C.^78,83^ The obtained QETD data allows one to estimate not only the total acid capacity as an amount of ammonia adsorbed in all temperature range but also amounts of weak and strong sites of various strength.

The acidity of all zeolites was investigated by adsorption of pyridine used as a probe molecule followed by FTIR spectroscopy. Generally studied zeolites were activated in a vacuum (5 × 10^−3^ torr) at 450 °C during 2 h. The adsorption and desorption of pyridine was carried out in hand-made IR cell with NaCl windows at temperature 150 °C (for selected samples – in the temperature range of 150–500 °C with step 50 °C) and investigated on a Spectrum One (PerkinElmer) spectrometer with a resolution of 1 cm^−1^. The concentration of Brønsted and Lewis type acid sites on Py-IR data was determined directly from the integral intensity of the corresponding bands in the IR-spectra (in adsorption mode) after adsorption of pyridine using the equations derived by Emeis.^86^

### 2.5. Catalytic measurement

The reaction of the ETBE synthesis was investigated in a flow reactor with a fixed bed. Ethanol was measured out from vessel standing under pressure 1.0 MPa with the help of dropwise sampler and “Whitey” needles. The space velocity of carrier gas (helium) was regulated by a needle and was fixed by a differential manometer. The temperature in the reactor was established and controlled by the controller/programmer type 812 “EUROTHERM” (Great Britain). The pressure in the reactor and in the vessel with ethanol was measured by model manometers.

Catalyst loading (volume of catalyst) was 1.0 cm^3^, grain size 0.25–0.50 mm. Molar ratio ethanol/isobutylene was 1.5, LHSV 1 h^−1^, carrier gas helium. The pressure was always 1.0 MPa. Studies were performed in the temperature range of 80–180 °C. Reaction products were amassed in a trap and analyzed by gas chromatograph Agat (“Mashpriborkomplekt”, Russia) equipped with Chromaton N-AW column with 10% Carbowax 600 (3 mm i.d., 2 m length) and a thermal conductivity detector.

The catalytic activity of zeolites for ETBE formation was characterized by the apparent ETBE formation rate, measured as the amount of ETBE formed per unit time per unit mass of catalyst (mol s^−1^ g^−1^).

## 3. Results and discussion

### 3.1. Structure and composition of zeolites H-BEA

**Fig. 1:**
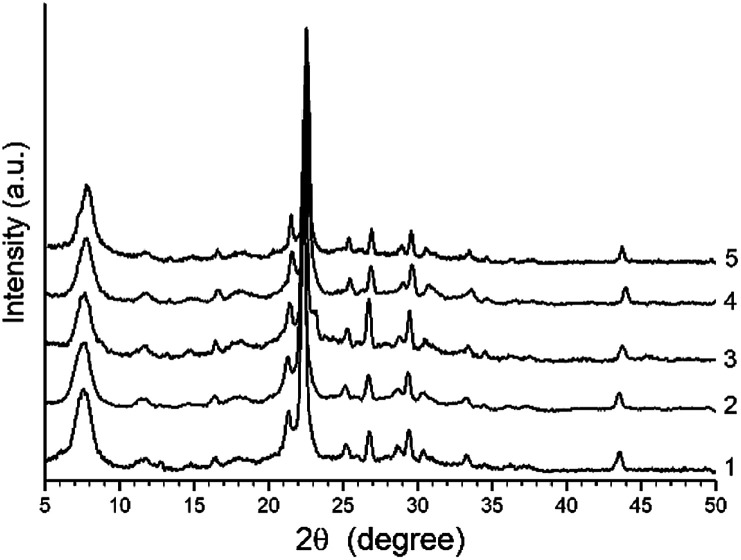
XRD patterns for H-BEA zeolites: (1) H-BEA 15, (2) H-BEA 32, (3) H-BEA 72, (4) H-BEA 124; (5) H-BEA 407.

The XRD patterns of H-BEA zeolites is shown in Fig. 1. The XRD patterns of all H-BEA samples are identical, indicating no structural changes under the Si/Al ratio variation within the studied range. All the solids are highly crystalline and no reflections other than those from BEA zeolite are observed. The XRD patterns of all zeolites match perfectly those reported in the literature for zeolite BEA.^87,88^

**Fig. 2:**
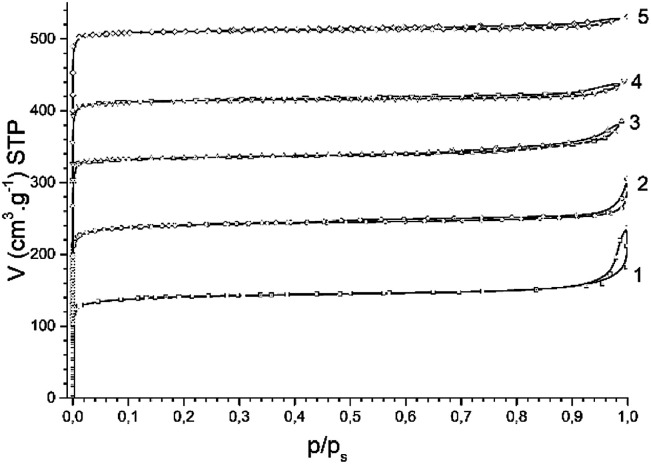
N_2_ adsorption isotherms for H-BEA zeolites: (1) H-BEA 15, (2) H-BEA 32, (3) H-BEA 72, (4) H-BEA 124; (5) H-BEA 407.

Nitrogen adsorption isotherms for all studied H-BEA zeolites are characteristic of a microporous material lacking secondary micropores and mesopores. The nitrogen adsorption isotherms of H-BEA zeolites is shown in Fig. 2.

The most part of nitrogen adsorption isotherms (up to *p*/*p*_s_ about 0.9) belongs to type I of IUPAC classification, which is characteristic of microporous adsorbents.^89^ Small hysteresis of type H3 together with increasing adsorption of nitrogen at high relative pressures with no limiting uptake is characteristic to aggregates of particles. The uptake of nitrogen at high relative pressures contains about one-third of total nitrogen adsorption for H-BEA samples. The composition of H-BEA samples and their textural properties are listed in Table 1.

**Table 1:** The composition and texture characteristics of H-BEA zeolites

Sample	Al content, *C*_Al_		Si/Al	*S*_BET_, m^2^ g^−1^	*V*_micro_, cm^3^ g^−1^
	mmol g^−1^	% wt			
H-BEA 15	1.06	2.87	14.8	558	0.21
H-BEA 32	0.50	1.35	32.4	612	0.21
H-BEA 72	0.23	0.62	71.5	632	0.20
H-BEA 124	0.13	0.36	123.9	646	0.20
H-BEA 407	0.04	0.11	407.4	670	0.19

The BET surface area for H-BEA samples is found in the range of 558–670 m^2^ g^−1^ and the microporous volume of crystalline zeolites samples is in the range of 0.19–0.21 cm^3^ g^−1^. The BET surface area shows a tendency to increase with decreasing the Al content, whereas the micropore volume slightly decrease.

### 3.2. Acid capacity and strength

**Fig. 3:**
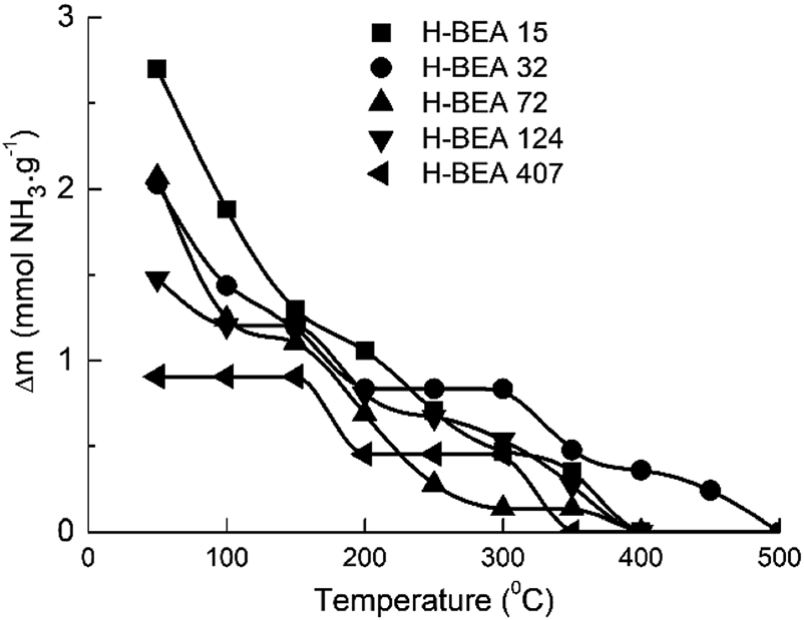
QETD-curves of ammonia thermal desorption from a surface of H-BEA zeolites.

**Fig. 4:**
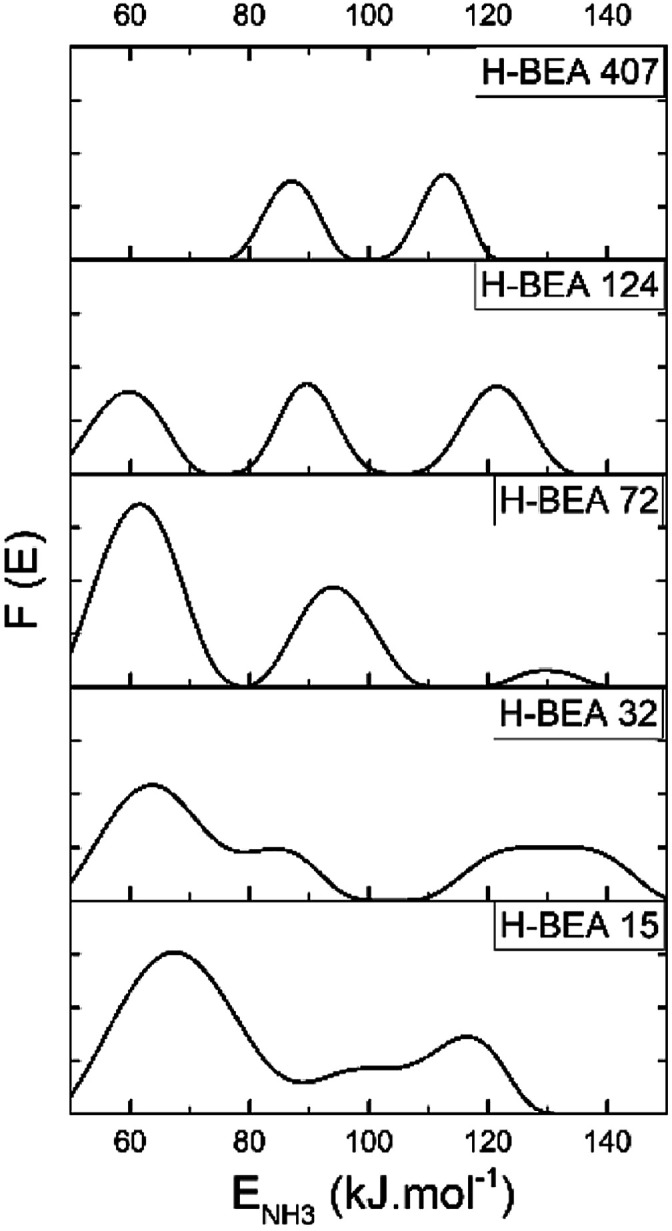
Ammonia adsorption energy distribution for H-BEA zeolites.

QETD curves of ammonia desorption for H-BEA samples are shown in Fig. 3. They have a complicated character with several areas of intensive weight loss due to the ammonia desorption and the horizontal sections where ammonia desorption is not observed. Using CONTIN method allows to calculate the ammonia adsorption energy for acid sites of studied zeolites.^78,83^ The corresponding ammonia adsorption energy distribution profiles for H-BEA samples are shown in Fig. 4, highlighting the energetic heterogeneity of acid sites. These profiles clearly show that generally the acidity spectrum of BEA zeolites includes three types of sites of different strengths: weak, medium, and strong. The data on the concentration and strength (ammonia adsorption energy) for each type of sites are presented in Table 2.

**Table 2:** Acid characteristics of H-BEA samples

Parameter	Acid sites	Si/Al				
		15	32	72	124	407
Aluminum content, mmol g^−1^		1.06	0.5	0.23	0.13	0.04
Acid capacity, mmol g^−1^	w	1.64	0.83	0.96	0.27	0.00
	m	0.59	0.36	0.97	0.54	0.45
	s	0.47	0.84	0.14	0.67	0.45
	Total	2.70	2.03	2.07	1.48	0.90
Average NH_3_ adsorption heat, kJ mol^−1^	w	68.9	64.8	61.6	59.6	—
	m	99.1	86.2	94.3	89.7	86.9
	s	115.9	130.0	129.8	121.4	112.4
	Zero coverage	130	150	142	133	120

Increasing the Si/Al ratio for H-BEA results in decreasing the total acid capacity, which is in a good agreement with a general tendency that acid capacity for zeolites, is caused by the presence of aluminum in their structure. However, it is worth noting that the total acid capacity of H-BEA samples determined by ammonia QETD significantly exceeds the corresponding value that may be calculated from the aluminum content as it follows from a comparison of the data presented in Tables 1 and 2. Therefore, a noticeable portion of acid sites in H-BEA is not related directly to the presence of aluminum in the zeolite framework. Probably, these weak acid sites correspond to silanol groups on the surface of the zeolite.

Our studies give the ammonia adsorption energy in the range of 59–69 kJ mol^−1^ for the weak acid sites which is close to the ammonia adsorption energy of 58.4 kJ mol^−1^ calculated for the silanol groups in silica.^90^ The higher ammonia adsorption heat for silanol groups of zeolites, compared with silanols in silica, may be associated with the induced action of aluminum.^21,91^ An increase of the aluminum content for H-BEA zeolite is accompanied by an increase in *E*_NH_3__ for weak acid sites as it follows from the data presented in Table 2.

According to the data presented in Table 2, the heat of ammonia adsorption at zero coverage is in the range of 120–150 kJ mol^−1^ which is in a good agreement with data obtained for zeolite H-BEA by a microcalorimetric method.^24^ The heat of ammonia adsorption at zero coverage depends nonmonotonically on the Si/Al ratio. It reaches the maximum value for H-BEA-32.

The same tendency also holds for the heat of ammonia adsorption at strong acid sites. This result is in agreement with previously reported data for the heat of ammonia adsorption dependence on Si/Al ratio for H-ZSM-5 zeolite that showed a maximum strength for Si/Al in the range between 18 and 35.^21,24^ The strength of medium acid sites changes nonmonotonically too. The strength of weak acid sites presented by the heat of the ammonia adsorption decreases slightly with increasing the Si/Al ratio. These sites are absent in zeolite H-BEA-407.

### 3.3. FTIR study

**Fig. 5:**
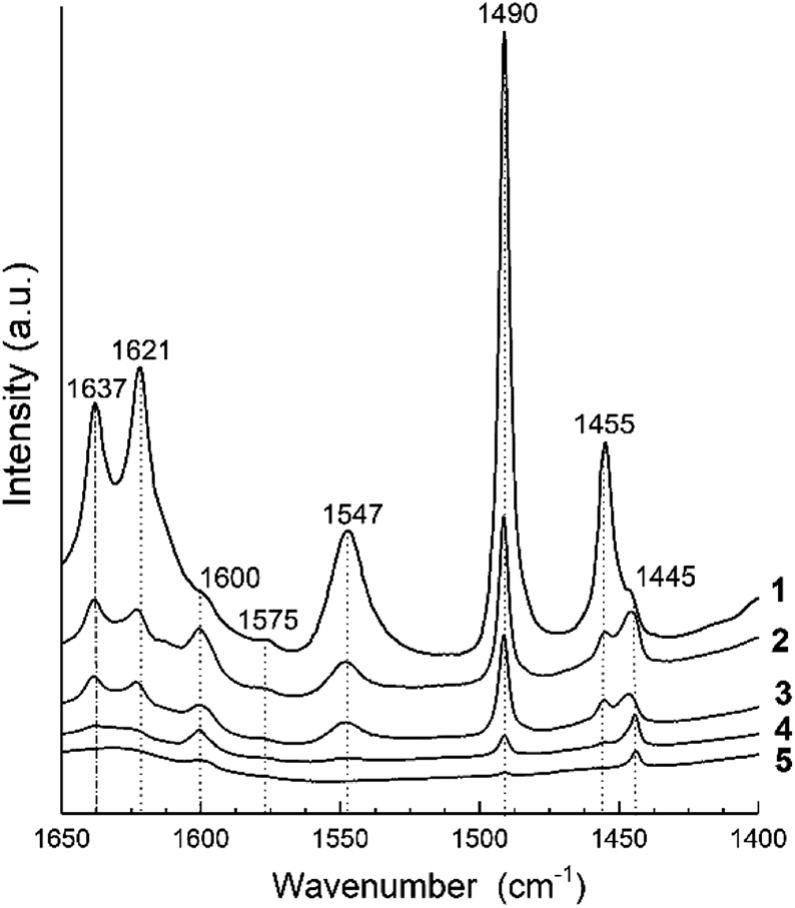
FTIR spectra of pyridine adsorption for H-BEA zeolites with various Si/Al ratio: (1) H-BEA 15, (2) H-BEA 32, (3) H-BEA 72, (4) H-BEA 124, (5) H-BEA 407.

The IR spectra of adsorbed pyridine for all samples in the 1400–1650 cm^−1^ range are shown in Fig. 5. They present the bands corresponding to formation of protonated, PyH^+^ (bands at 1637, 1490 and 1547 cm^−1^), coordinated, PyL (bands at 1621, 1490, 1445 and 1455 cm^−1^) and H-bonded (bands at 1600 and 1575 cm^−1^) pyridine species.^32,92,93^ According to^94^ the band at 1445 cm^−1^ can be assigned to pyridine bonded to a relatively stronger Lewis site formed on three-coordination or extra-framework Al species. The band centered at 1490 cm^−1^ displays pyridine adsorbed on both the Lewis and Brønsted acid sites.

**Fig. 6:**
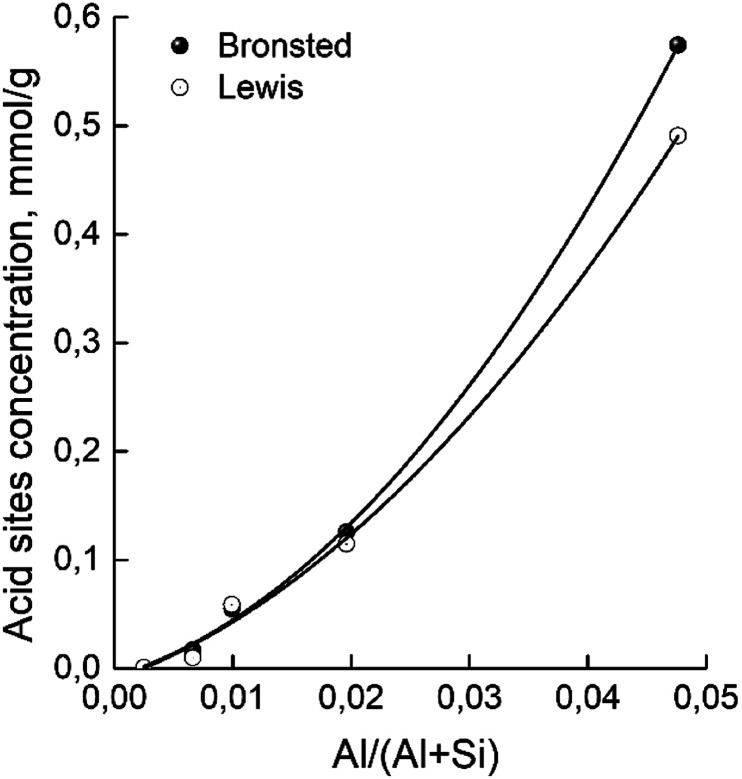
Dependence of concentrations of Brønsted and Lewis acid sites of H-BEA zeolites on aluminum relative content.

The concentrations of Brønsted and Lewis acid sites were calculated based on the published extinction coefficients^86^ for the bands at 1545 cm^−1^ and, 1455 cm^−1^ correspondingly. Fig. 6 gives these concentrations as a function of the aluminum relative content in zeolites H-BEA. Increasing aluminum content increases the concentrations of both Brønsted acid sites and Lewis acid sites.

**Fig. 7:**
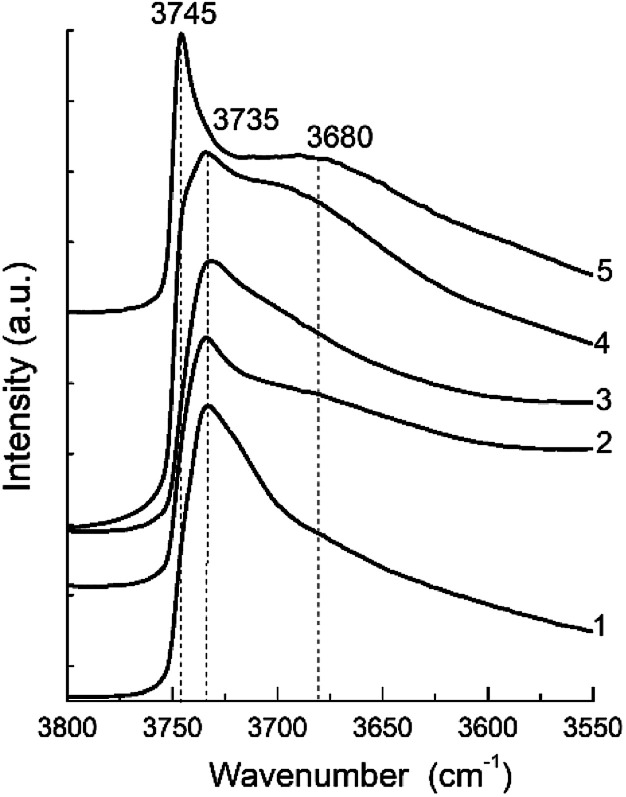
FTIR spectra of the hydroxyl region of the zeolite H-BEA samples with various Si/Al ratio: (1) H-BEA 15, (2) H-BEA 32, (3) H-BEA 72, (4) H-BEA 124; (5) H-BEA 407.

The FTIR spectra in the hydroxyl range for different samples are show in Fig. 7. They present a clearly distinguishable bands at 3745 and 3735 cm^−1^ and a broad shoulder about 3680 cm^−1^.

The most intensive bands observed at 3745 and 3735 cm^−1^ may be attributed to external and internal silanol groups respectively. The band at 3735 cm^−1^ corresponds to silanol groups localized inside the zeolite cavities, and the band 3745 cm^−1^ corresponds to terminal silanol groups located on the exterior surface.^33,95^ The band centered at 3680 cm^−1^ assigned to amorphous extra-framework A1–O–OH species.^95^

The IR spectra for the most of studied zeolites contain clearly resolved band at 3735 cm^−1^. The intensity of this band decreases with an increase in the Si/Al ratio up to 124. Contrary to all other samples, the band at 3735 cm^−1^ disappears in the FTIR spectra for H-BEA-407, whereas band at 3745 cm^−1^ is clearly resolved. Therefore, exterior surface and it does not contain internal silanol groups localized inside zeolite cavities. The band at 3745 cm^−1^ is also presented as a shoulder in the spectrum of H-BEA-124. The small broad shoulder in FTIR spectra in the region between 3600 and 3700 cm^−1^ is probably associated with OH groups bonded to extra-framework aluminium.^95–97^ These groups are presented in the samples with high Si/Al ratio (H-BEA-124 and H-BEA-407).

### 3.4. ^27^Al MAS NMR study

**Fig. 8:**
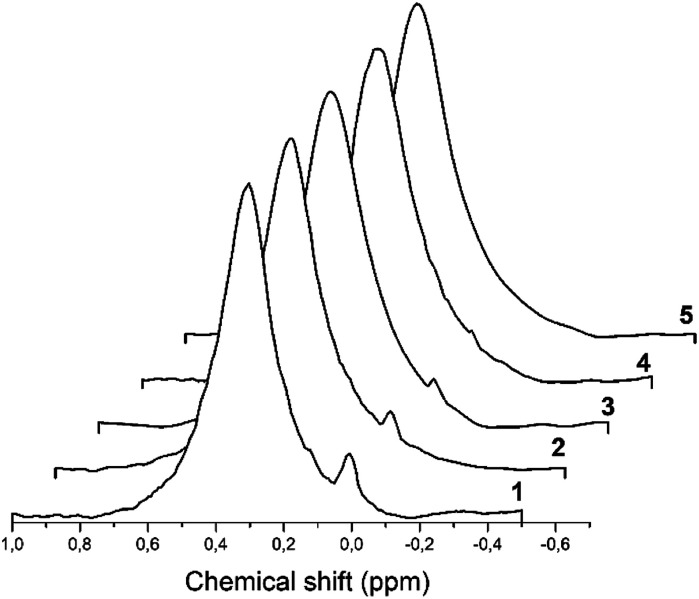
The ^27^Al MAS spectra demonstrate a signal of octahedrally coordinated aluminum detected in the H-beta-zeolites: 1–H-BEA 15; 2–H-BEA 72; 3–H-BEA 407.

^27^Al MAS spectra of all hydrated solids are presented in Fig. 8 where intensity is given in arbitrary units.

For almost all samples, ^27^Al MAS NMR spectra are characterized by two main signals at ∼54–58 and 0 ppm which may be assigned to tetrahedral and octahedral Al atoms, respectively.^98–100^ The broad peak at 55 ppm may be attributed to the distorted tetra-coordinated framework aluminum as a result of strong quadrupole interactions.^101–103^ NMR spectra also contain a small signal at around 0 ppm, associated with the octahedrally coordinated extra-framework aluminum.^99,104^Fig. 8 shows that the band of 0 ppm, corresponding to octahedrally coordinated aluminum, present in all samples except H-BEA 407.

### 3.5. ETBE synthesis over H-BEA zeolites

**Fig. 9:**
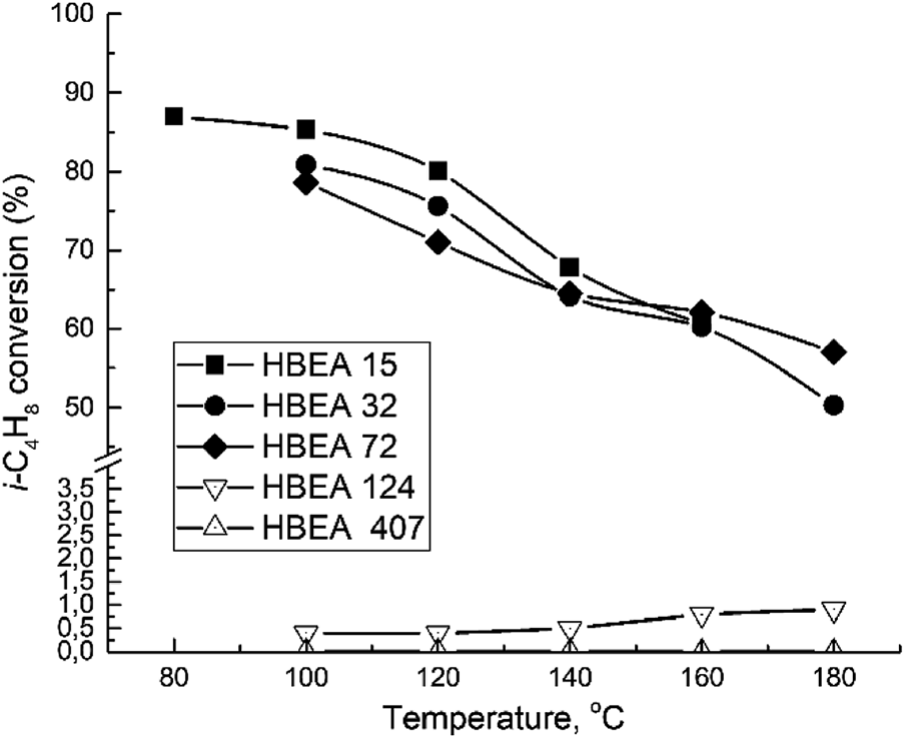
Temperature dependence of i-C_4_H_8_ conversion for H-BEA zeolites.

**Fig. 10:**
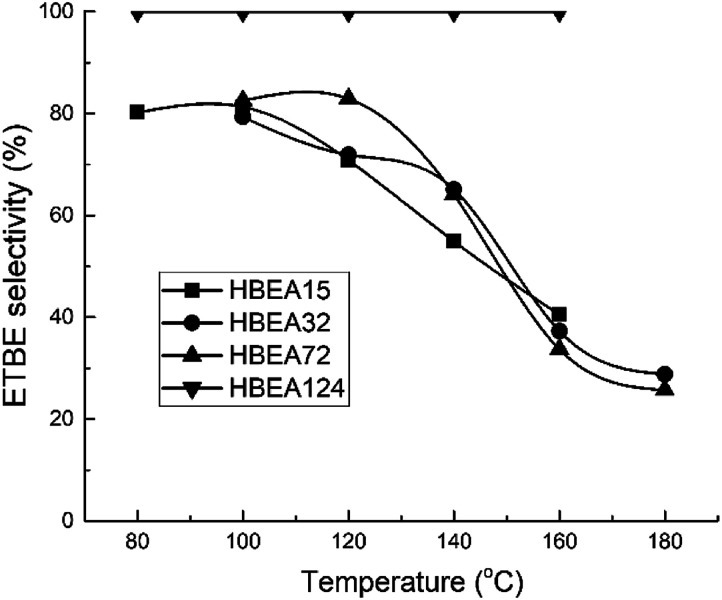
Temperature dependence of ETBE selectivity for H-BEA zeolites.

All samples show catalytic activity in the ETBE synthesis except H-BEA-407. The products of the catalytic transformations of ethanol and isobutylene over H-BEA zeolites are ETBE and *tert*-butyl alcohol (*t*-BuOH). Appearance of *t*-BuOH in reaction products is a consequence of the water present in the reaction mixture as a part of the azeotropic ethanol-water mixture. At temperatures above 120 °C, over H-BEA 32 and H-BEA 72, the diethyl ether (DEE) and di-isobutylene formation is observed. Fig. 9 and 10 shows the temperature dependences of iso-butylene conversion and ETBE selectivity for studied H-BEA samples. Zeolite H-BEA-15 shows the highest catalytic activity, whereas zeolite H-BEA-124 exhibits very low activity. It is worth noting that zeolite H-BEA-407 does not show any catalytic activity for all temperature range.

Catalytic behavior of zeolites H-BEA significantly depends on the Si/Al ratio. Increasing the Si/Al ratio results in decreasing the iso-butylene conversion that is accompanied by an increase in the optimal temperature of the process. Increasing temperature above 100–120 °C decreases the i-C_4_H_8_ conversion due to the thermodynamics limitations resulting from the process exothermicity.^101^ For samples of H-BEA 15, H-BEA 32 and H-BEA 72 the ETBE selectivity coincides closely. Wherein, ETBE selectivity sharply decreases above 120 °C due to the contribution of the side reactions of DEE and di-isobutylene formation. The low-active H-BEA-124 catalyst is characterized by the 100% ETBE selectivity; however, the conversion values for this sample do not exceed 1%.

**Fig. 11:**
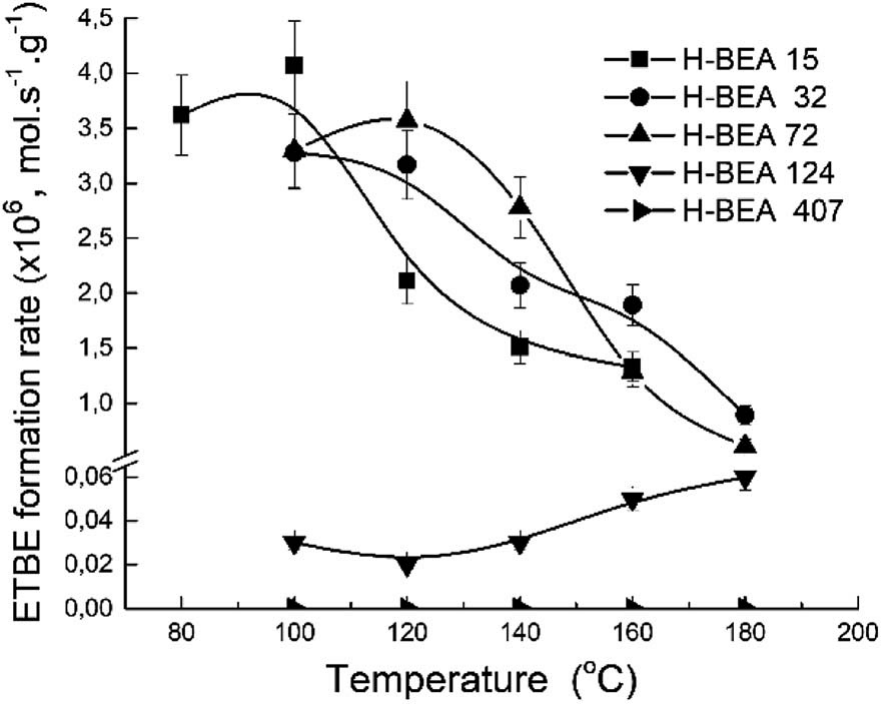
Temperature dependence of apparent ETBE formation rate for zeolites H-BEA.

The porous structure of the zeolite catalysts is almost the same as it follows from the XRD studies and nitrogen adsorption data. The catalytic experiments were carried out under the same conditions for each sample. Therefore, the apparent ETBE formation rate (mol s^−1^ g^−1^) gives a comparative characterization of the catalytic activity of the studied samples. Fig. 11 shows a temperature dependence of the apparent ETBE formation rate for the studied samples.

### 3.6. A relation between acid and catalytic properties for zeolites H-BEA

**Fig. 12:**
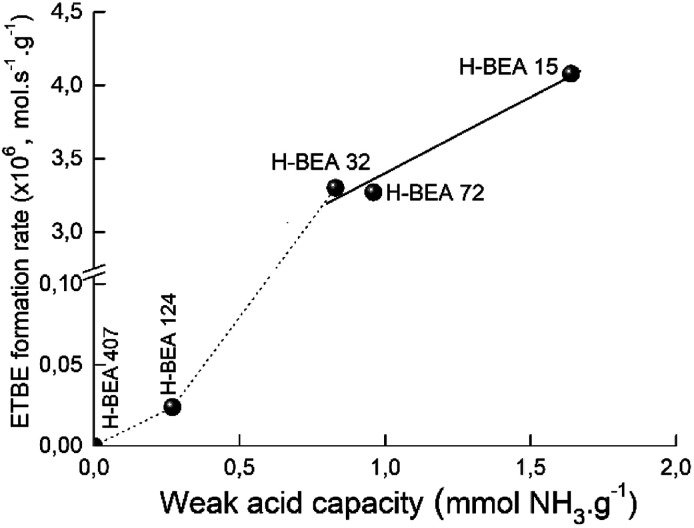
Correlation of apparent ETBE formation rate on the concentration of weak acid sites of zeolites H-BEA.

Fig. 12 gives the dependence of the apparent ETBE formation rate on the concentration of w-sites for zeolites H-BEA. The catalytic activity and acidity of H-BEA zeolites were compared at the temperature of 100 °C because at this temperature only one side reaction, the *tert*-butanol formation, takes place. At higher temperatures, the reaction of the ETBE formation follows the thermodynamic equilibrium.^105,106^

A linear correlation is observed for zeolites H-BEA-15, H-BEA-32, and H-BEA-72, *i.e.* for zeolites with low Si/Al ratio. Increasing the Si/Al ratio results in a sharp falling down the activity for zeolites H-BEA-124 and H-BEA-407.

Fig. 12 illustrates that catalytic activity increases with increasing the concentration of the weak acid sites. The data presented in Table 2 show that increasing the concentration of the weak acid sites is accompanied by an increase in the total concentration of acid sites. As a result, the apparent ETBE formation rate of zeolites H-BEA in the ETBE synthesis increases with increasing the total concentration of acid sites. However, this is just a coincidence resulted from similar dependencies of both, apparent ETBE formation rate and the total concentration of acid sites, on the concentration of the weak acid sites. Our results show the absence of the catalytic activity for the sample H-BEA-407 which does not contain the weak acid sites.

### 3.7. The nature of active sites of zeolite H-BEA

A correlation between the catalytic activity and the concentration of weak acid sites supports that the weak acid sites are mainly responsible for the catalytic performance of zeolites H-BEA. Catalytic studies illustrate that zeolite H-BEA-407 does not show catalytic activity. According to the QETD studies, zeolite H-BEA-407 does not contain weak acid sites as it follows from the data presented in Table 1. Therefore, the catalytic activity of zeolites H-BEA in the isobutylene etherification with ethanol is associated with the weak acid sites. The ammonia QETD studies show that zeolites H-BEA may contain three types of acid sites, which are typical for zeolites.^21,83^ The strong acid sites, apparently, can be assigned to the Lewis sites representing three-coordinated aluminum atoms. The sites of medium strength can be assigned to the “classic” Brønsted acid sites represented by the bridging hydroxyl groups, Si(OH)Al.^21,107^ The nature of weak acid sites is less clear. For the weak acid sites, our data give the ammonia adsorption energy in the range of 59–69 kJ mol^−1^. The range of the ammonia adsorption energy is in agreement with the reported value of 62 kJ mol^−1^ for the Brønsted acid sites of zeolite H-BEA.^108^ Therefore, we may assume that the weak acid sites are associated with hydroxyl groups.

Comparison of the QETD and FTIR studies for zeolites H-BEA suggests that the band at 3735 cm^−1^ corresponds to weak acid sites which are presented in all samples except H-BEA-407 as it follows from the data presented in Table 2. According to data presented in the literature,^33,95^ the band at 3735 cm^−1^ corresponds to silanol groups localized inside the zeolite cavities, *i.e.* to internal silanol groups. It has been reported that the internal silanol groups display a significant Brønsted acidity, which is higher compared to the acidity of silanol groups located at the external crystal surface.^33,97,109^ Moreover, acidic properties of the surface functional groups are dependent on the nature of the neighboring atoms, and even atoms of the second coordination environment.^24^ The proton mobility in silanol groups may be enhanced by the presence of aluminum in zeolite structure. The presence of aluminum in the first coordination sphere of silanol group forms strong bridging Brønsted site. If it presents in the second coordination sphere, it also affects the proton mobility in silanol group. As a result, the corresponding silanol group exhibits properties of a weak Brønsted acid site.

**Scheme 1:**
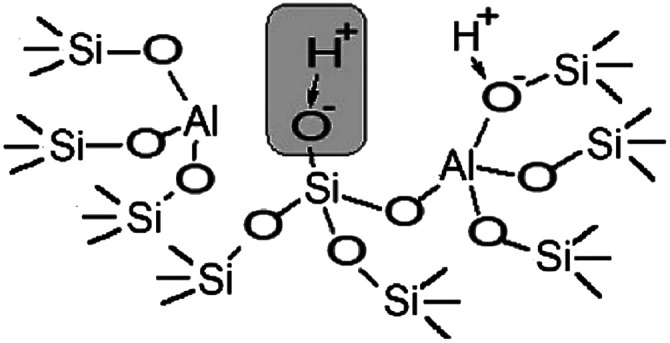
Schematic drawings of the structure of weak acid sites of H-BEA zeolites.

**Scheme 2:**
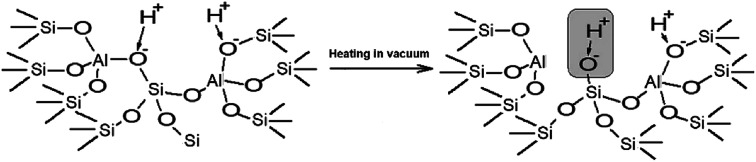
Schematic drawings of the formation of weak acid sites of H-BEA zeolites.

The performed studies allow us to suggest the structure of the active site which is responsible for the catalytic activity of zeolites H-BEA in the isobutylene etherification with ethanol. This structure is shown in Scheme 1. This weak Brønsted acid site represents a silanol group with tetrahedral aluminum in the second coordination sphere. Its occurrence is accompanied by the formation of octahedral aluminum species. These structures can form by the partial hydrolysis of Si–O–Al bonds during the calcination, when the strong Brønsted acid site (bridging OH group) breaks up into the Lewis acid site (tri-coordinated aluminum) and the weak Brønsted acid site (Si–OH group).^100,110,111^ This process is illustrated in Scheme 2.

We note the corresponding samples contain octahedral aluminum what is in a good agreement with our ^27^Al MAS NMR studies that showed the presence of octahedral aluminum in all samples except catalytically inactive H-BEA-407.

**Fig. 13:**
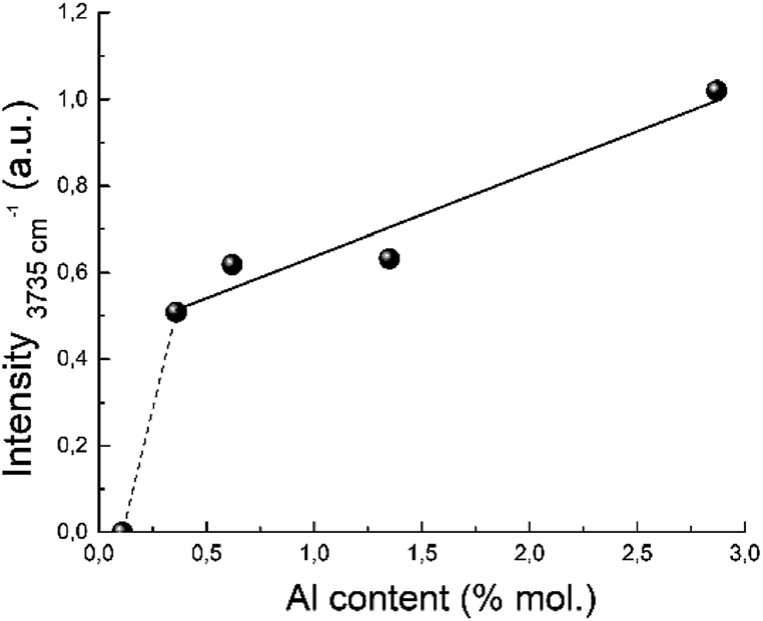
Correlation between the 3735 cm^−1^ IR band intensity and the aluminum content.

The proposed structure of the active site is also in a good agreement with the IR study. The band near 3735 cm^−1^ corresponds to OH groups bonded to Si atoms linked to three-coordinated Al atoms.^97^ Its integral intensity correlates with the aluminum content (see Fig. 13). The linear correlation between the band intensity at 3735 cm^−1^ and the concentration of aluminum is observed for the all zeolites with Si/Al ratio up to 124. This band corresponding to internal silanol groups totally disappear for zeolites with low aluminium content, Si/Al > 124.

A disappearance of the band at 3735 cm^−1^ in IR spectra at Si/Al ratio less than 124 can be explained from the NMR data.^100^ It has been shown that the amount of octahedral aluminium species decreases with increasing Si/Al ratio and octahedral aluminium was absent for the high-silica H-BEA samples. According to the Loewenstein's rule, two aluminium sites, which are adjacent or close to each other, are required for the hydrolysis of a Si–O–Al bond for the formation of octahedrally coordinated aluminium. Therefore, observed dependence (Fig. 13) can confirm the identification of the absorption band of 3735 cm^−1^ with the acid sites associated with octahedral aluminum.

**Fig. 14:**
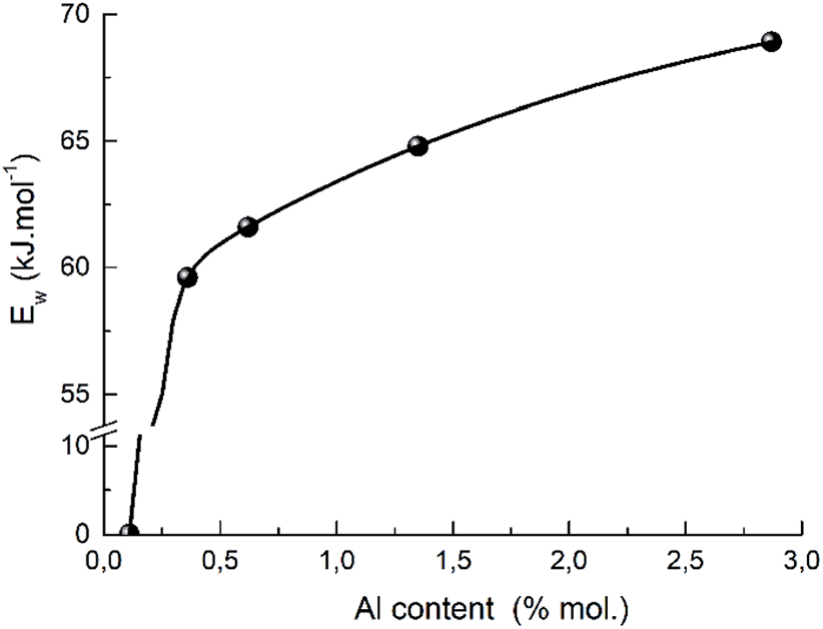
The dependence of the strength of weak acid sites on aluminum content in H-BEA zeolite.

A presence of the aluminum also promotes the proton mobility which shows the correlation between the strength of weak acid sites and the aluminum content in H-BEA zeolites (Fig. 14). A similar effect was recently reported for ZSM-5 zeolites with varying Si/Al ratio.^112^ The observed correlation also supports the proposed structure of weak acid sites of zeolites H-BEA illustrating the aluminium effect on a sharp decrease in strength of the weak acid sites with decreasing the aluminium content in the zeolite.

Decreasing the aluminum content results in lowering a probability to find two adjacent or closely located aluminum atoms in the zeolite structure. Consequently, the proposed structure of the active site cannot be realized if the aluminum content is too small.^100^

A formation of the weak acid sites with the discussed structure is accompanied by a formation of three-coordinated aluminium species. According to a number of works^103,111,113^ a signal at 0 ppm in NMR spectra associated with the octahedral aluminum in zeolites structure represents an extra-framework aluminum. Our suggestions about the formation of active sites of H-BEA zeolites allows us to speculate on the role of the extra-framework aluminum in catalytic behavior of zeolites which has been discussed in several publications and still did not find a logical explanation. Studies have shown a positive effect of the extra-framework aluminium on the catalytic activity of zeolites. However, a pronounced correlation was not found between the catalytic activity and the concentration of the extra-framework aluminium.^114–116^ We suggest that the extra-framework aluminum is a “by-product” of the formation of proposed weak acid sites. As a result, the apparent catalytic activity may be associated with a presence not of the extra-framework aluminum but with the silanol groups in a specific environment. These silanol groups are responsible for the weak Brønsted acidity and for the catalytic activity of zeolites in the etherification of isobutylene with ethanol.

## 4. Conclusions

In the present work, we determined the nature of the acid sites of the H-BEA zeolites responsible for the heterogeneous catalytic synthesis of ethyl *tert*-butyl ether from ethanol and isobutylene. QETD and catalytic studies emphasize that etherification of isobutylene with ethanol over zeolites occurs with the participation of weak Brønsted acid sites, which are characterized by an ammonia adsorption energy in the range of 59–69 kJ mol^−1^. The adsorbed pyridine FTIR studies and NMR data suggest that these weak acid sites are identified as the Brønsted hydroxyls representing internal silanol groups. We concluded that the structure of the active site forms by the partial hydrolysis of the Si–O–Al bonds during the zeolite calcination and accompanied by the formation of particles of three-coordinated aluminum. This is proved by the correlation of absorption band 3735 cm^−1^ intensity in FTIR spectrum with the aluminum content. The aluminum content also has an impact on the strength of weak acid sites. The disappearance of the weak acid sites for zeolites H-BEA with low aluminum content is associated with the absence of two nearby aluminum atoms in zeolite framework. Hence, the disruption of linear correlation of catalytic activity and weak acidity for high-silica H-BEA zeolites results from the difficulty of formation of internal silanol groups at low aluminum content. Our correlations show an importance of the Brønsted weak acid sites in the catalytic performance of zeolites. The weak acid silanol groups may play an important role not only in the etherification reactions but also in the alkylation and acylation processes, which occur under soft conditions and do not require a break of chemical bonds in molecules.

## Conflicts of interest

There are no conflicts to declare.

## Supplementary Material
